# CTLA-4、PD-1和PD-L1在小细胞肺癌外周血中的分布及临床意义

**DOI:** 10.3779/j.issn.1009-3419.2017.11.06

**Published:** 2017-11-20

**Authors:** 慧 李, 岩 刘, 影 柳, 菁菁 柳, 丹丹 赵, 莹 王, 颖 程

**Affiliations:** 1 130012 长春，吉林省肿瘤医院肿瘤转化医学实验室 Medical Oncology Translational Research Lab, Jilin Cancer Hospital, Changchun 130012, China; 2 1 130012 长春，吉林省肿瘤医院胸部肿瘤内科 Department of Medical Thoracic Oncology, Jilin Cancer Hospital, Changchun 130012, China

**Keywords:** CTLA-4, PD-1, PD-L1, 小细胞肺癌, 生物标志物, CTLA-4, PD-1, PD-L1, SCLC, biomarker

## Abstract

**背景与目的:**

本研究旨在探索细胞毒性T淋巴细胞相关抗原（cytotoxic T lymphocyte associated antigen-4, CTLA-4）、程序坏死因子（programmed death 1, PD-1）和程序坏死因子配体（programmed death ligand 1, PD-L1）在小细胞肺癌（small cell lung cancer, SCLC）患者外周血中的分布情况，探索其免疫作用机制并评估其作为生物标志物的临床价值。

**方法:**

招募290例SCLC患者及60例健康志愿者，收集治疗前和治疗2^nd^周期末SCLC患者EDTA抗凝血2 mL。应用流式细胞仪检测CTLA-4、PD-1和PD-L1在外周血CD3、CD4、CD8及CD25的分布，分析其与临床病理特征的相关性；采用细胞免疫化学法和流式细胞法检测PD-L1在SCLC细胞系H446中的表达。

**结果:**

SCLC患者外周血中CTLA-4^+^细胞和PD-1^+^细胞水平分别为（1.56±1.24）%和（8.07±3.97）%、CTLA-4在CD3细胞和CD4细胞中的表达水平无明显差异，分别为（4.87±5.18）%和（3.85±2.60）%，均低于PD-1在CD3^+^或CD4^+^细胞中的表达（26.63±9.04）%和（20.79±9.41）%，与健康对照组相比，SCLC中CD4^+^CD25^+^CTLA-4^+^细胞水平明显升高（1.91±1.27）% *vs*（7.09±5.09）%，*P* < 0.001；PD-1^+^（CD8^+^）细胞表达水平明显降低，分别为（22.56±4.21）% *vs*（11.47±5.85）%，*P* < 0.001。CD4^+^CD25^+^CTLA-4^+^细胞或CD8^+^PD-1^+^细胞水平与患者的年龄、性别、吸烟状况、临床分期以及是否转移等因素无关（*P* > 0.05）。化疗两周期末CD4^+^CD25^+^CTLA-4^+^和CD8^+^PD-1^+^细胞的水平对比化疗前明显下降，分别为（5.11±2.60）% *vs*（6.94±4.91）%；（8.74±3.39）% *vs*（11.48±5.91）%，*P*值均 < 0.000, 1，但与无疾病进展生存和总生存无显著相关性。PD-L1高表达于SCLC细胞系H446中并定位在细胞膜和细胞浆，但在外周血中未见表达。

**结论:**

本研究首次证实SCLC外周血中CTLA4高表达于调节性T细胞中，而PD-1低表达于效应性T细胞，该结果为揭示SCLC免疫监测点免疫逃逸机制提供了理论依据，可能作为一种新的无创性且可实时监测的生物标志物。

## Abstract (en)

**Background and objective:**

The aim of this study is to explore cytotoxic T lymphocyte associated antigen 4 (CTLA-4), programmed death 1 (PD-1) and programmed death ligand 1 (PD-L1) distribution and clinical value in liquid biopsy (such as blood) of small cell lung cancer (SCLC) patients.

**Methods:**

A total of 60 healthy and 290 chemotherapy-naive patients with SCLC were recruited. Venous blood samples were collected prior to chemotherapy (baseline) and after the second cycle of chemotherapy (2^nd^ cycle), and flow cytometry was used to analyze the level of CTLA-4, PD-1 or PD-L1 with or without CD3, CD4, CD8 or CD25. Immunocytochemical method was used to detect PD-L1 expression in SCLC cell line H446.

**Results:**

Cells of CTLA-4^+^ and PD-1^+^ in SCLC peripheral blood were (1.56±1.24)% and (8.07±3.97)%; there is no significant difference between CD3^+^CTLA-4^+^ and CD4^+^CTLA-4^+^, (4.87±5.18)% and (3.85±2.60)%, but show lower expression than CD3^+^PD-1^+^ and CD4^+^PD-1^+^ (26.63±9.04)% and (20.79±9.41)%, respectively. However, the level of CD4^+^CD25^+^CTLA-4^+^ cells were remarkably higher in SCLC than that in control, (7.09±5.09)% *vs* (1.91±1.27)%, *P* < 0.001 and CD8^+^PD-1^+^ cells were less in SCLC than that in control, (11.47±5.85)% *vs* (22.56±4.21)%, *P* < 0.001, both of which were not associated with age, sex, smoke or disease stage. Level of CD4^+^CD25^+^CTLA-4^+^ cells and CD8^+^PD-1^+^ cells was dropped (5.11±2.60)% *vs* (6.94±4.91)% and (8.74±3.39)% *vs* (11.48±5.91)% after 2^nd^ cycle compare to that at baseline (*P* < 0.000, 1). Neither the level of CD4^+^CD25^+^CTLA-4^+^ nor CD8^+^PD-1^+^ cells before or after treatment was related to progression-free disease or overall survival of patients. Although PD-L1 was highly expressed in H446 cell cytoplasm and membrane, it was rarely found in peripheral blood.

**Conclusion:**

The data we presented here showed that CTLA-4 was highly expressed in regulatory T cells and PD-1 decreased in CD8^+^ T cells in peripheral blood of SCLC patients, suggesting their unique mechanisms involved in immune regulation. CD4^+^CD25^+^CTLA-4^+^ level changed after treatment implies its potential role in predicting treatment efficacy.

肺癌是影响国民健康的头号杀手，发病率和死亡率均居恶性肿瘤的首位，其中小细胞肺癌（small cell lung cancer, SCLC）约占肺癌总数的10%-15%。可进行手术切除的小细胞肺癌患者比例低，大部分患者需接受药物治疗，化疗为主要治疗药物^[1]^。尽管SCLC患者对初始化疗敏感，但易复发，可选择的二线治疗药物较少，其耐药机制不明。SCLC的疾病发生和进展机制复杂，尽管普遍存在*Tp53*和*RB*基因等缺失，但缺少有效的靶向药物。近20年来SCLC的临床治疗缺乏突破，急需寻找新的治疗靶点和药物疗效预测标志物来改善SCLC的治疗现状^[2]^。

肿瘤免疫治疗成为肿瘤治疗的一个重要研究领域，细胞毒性T淋巴细胞相关抗原（cytotoxic T lymphocyte associated antigen-4, CTLA-4）、程序坏死因子（programmed death 1, PD-1）和程序坏死因子配体（programmed death ligand 1, PD-L1）的单药或联合用药已经获批用于临床治疗黑色素瘤、肾癌及非小细胞肺癌。在小细胞肺癌中，也有众多免疫靶向治疗临床研究正在进行。然而免疫靶向药物至今缺少疗效预测的生物标志物，因此难以挑选适合靶向治疗的人群，导致治疗缺乏针对性，总体疗效差^[3, 4]^。

CTLA-4、PD-1和PD-L1高表达于肿瘤浸润淋巴细胞或肿瘤中^[5-11]^，众多研究将其作为肿瘤免疫靶向的疗效预测标志物，但研究结果受标本、检测方法、结果判读标准的限制，目前缺乏统一共识。SCLC组织标本获取困难，液态标本具有实时无创的特点，可作为SCLC生物标志物检测的有效替代标本，本研究拟检测SCLC患者外周血中PD-1、PD-L1及CTLA-4在淋巴细胞中的分布情况，探讨其致病机制，分析上述生物标志物的临床价值，为SCLC免疫靶向药物的临床实践提供理论依据。

## 1 资料与方法

### 1.1 研究对象

招募吉林省肿瘤医院2014年1月-2016年1月住院并确诊为SCLC患者290例，其中男性176例，女性114例；广泛期114例，局限期176例；年龄39岁-71岁，中位年龄51岁。所有患者均未进行放化疗或免疫治疗，另外，选取60名健康志愿者作为对照组，男性33例，女性27例，年龄范围30岁-60岁，中位年龄47岁。本研究已通过吉林省肿瘤医院医学伦理委员会批准，并签署知情同意书。

### 1.2 标本采集

所有SCLC患者（采血点：化疗前及化疗2周期末）和健康志愿者均分别抽取外周静脉血2 mL，4 ℃保存，2 h内检测。

### 1.3 试剂与仪器

人SCLC细胞系H446和前列腺癌细胞系PC-3购自美国ATCC细胞库，胎牛血清、DMEM培养液和胰酶均购自美国Hyclone公司；鼠抗人PE标记的PD-1/PD-L1单克隆抗体（CD279-PE/CD274-PE）、APC标记的CTLA-4单克隆抗体（CD152-PerCP）及鼠抗人CD3、FITC标记的鼠抗人CD4和CD25、PerCP标记的鼠抗人CD8单克隆抗体（CD8-PerCP）及流式细胞仪均购自BD公司。免疫化学染色试剂均购自中杉公司。

### 1.4 细胞培养

采用DMEM（含10% FBS）培养H446细胞和PC-3细胞，在37 ℃、5%CO_2_孵箱内培养，细胞达到90%覆盖率时进行细胞传代培养。

### 1.5 流式细胞仪检测

100 μL抗凝血中分别加入5 μL抗体，室温孵育30 min；应用200 μL红细胞裂解液裂解红细胞，涡旋振荡30 s，待抗凝血颜色澄清后立即以2, 000 rpm速度离心10 min，去除上清液，加入500 μL PBS重悬细胞沉淀，使用流式细胞仪上机检测。

### 1.6 免疫细胞化学检测蛋白表达

对数期生长的细胞消化后爬片培养24 h，4%多聚甲醛固定细胞，滴加一抗（1:200）湿盒中4 ℃冰箱孵育过夜。PBS洗涤3次后，滴加反应液（第二抗体），室温下温育30 min，DAB试剂盒显色5 min-10 min或显微镜下观察棕黄色反应后终止。流水冲洗后苏木素复染，常规树脂封片。PC-3细胞做阴性对照。

### 1.7 统计学分析

采用SPSS 17.0统计软件分析数据，计量资料以均数±标准差（Mean±SD）表示，组间差异比较应用*t*检验，以*P* < 0.05为差异有统计学意义。

## 2 结果

### 2.1 SCLC外周血中CTLA-4在调节性T细胞中高表达

在淋巴细胞、成熟T淋巴细胞（CD3阳性）和辅助性T淋巴细胞（CD4阳性）中，CTLA-4阳性细胞分别为（1.56±1.24）%、（4.87±5.18）%和（3.85±2.60）%，与健康对照组相比无明显差异（*P* > 0.05）。在调节性T细胞（CD4和CD25阳性）中，CTLA-4阳性细胞明显高于健康对照组（7.09±5.09）% *vs*（1.91±1.27）%，*P* < 0.001，提示SCLC免疫系统中的调节性T细胞可能通过高表达CTLA-4进一步发挥免疫抑制功能，从而帮助肿瘤实现免疫逃逸（图 1）。

**1:**
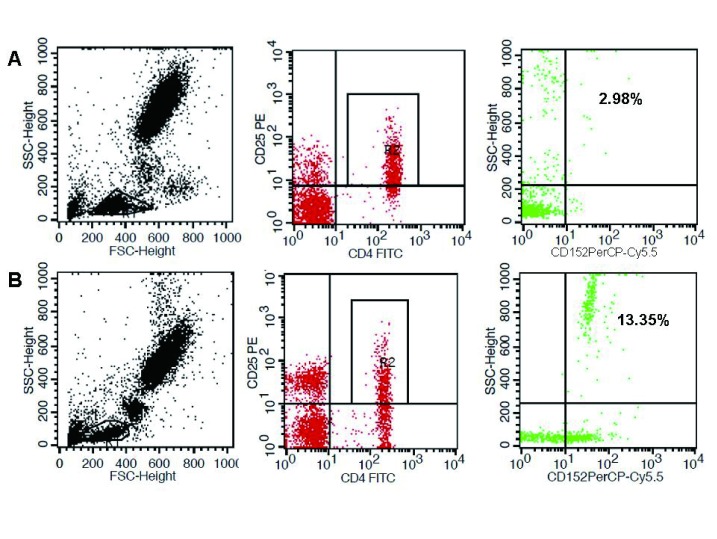
CTLA-4在外周血淋巴细胞中CD4^+^CD25^+^上的分布。A：健康对照组；B：SCLC组。 Distribution of CTLA-4 in CD4^+^CD25^+^ T cells in peripheral blood specimens. A: Healthy control group; B: SCLC group.

### 2.2 PD-1在外周血效应性T细胞（CD8^+^ T）中的分布表达

我们首先检测了SCLC患者外周血中总淋巴细胞和T细胞（PD-1^+^和CD3^+^PD-1^+^）中阳性细胞表达，分别为（8.07±3.97）%和（26.63±9.04）%，对比健康对照组无统计学意义（*P* > 0.05）。进一步检测辅助性T细胞（CD4）和效应性T细胞（CD8）中PD-1的表达水平后发现，与健康人相比，PD-1在效应性T细胞中表达水平明显降低，分别为（22.56±4.21%）*vs*（11.47±5.85）%，*P* < 0.001，但在辅助性T细胞中无显著差别，提示PD-1信号通路主要抑制效应性T细胞而不是辅助性T细胞（图 2）。

**2:**
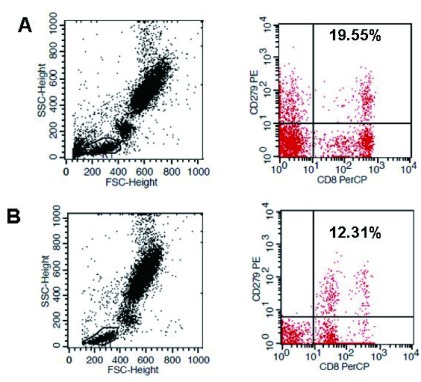
PD-1在CD8^+^ T细胞中的分布情况。A：健康对照组；B：SCLC组。 Distribution of PD-1 in CD8^+^ T cells in peripheral blood. A: Healthy group; B: SCLC patients.

### 2.3 SCLC外周血中PD-1及CTLA4与临床病理因素的关系

CD4^+^CD25^+^CTLA-4^+^细胞和CD8^+^PD-1^+^细胞水平与患者的年龄、性别、吸烟状况、临床分期以及是否转移等因素无关（*P* > 0.05）（表 1）。但经2周期化疗后CD4^+^CD25^+^CTLA-4^+^及CD8^+^PD-1^+^的表达水平比治疗前降低，差异具有统计学意义，分别为（6.94±4.91）% *vs*（5.11±2.60）%和（11.48±5.91）% *vs*（8.74±3.39）%，*P*均 < 0.000, 1（表 2）。

**1:** SCLC患者外周血中CD8^+^PD-1^+^及CD4^+^CD25^+^CTLA-4^+^与临床病理因素的关系 The relationship between CD8^+^PD-1^+^ and CD4^+^CD25^+^CTLA-4^+^ level and clinicopathologic parameters in the patients with SCLC

Pathologic parameter	*n*	PD-1 (%)	*P*	CTLA-4 (%)	*P*
Gender			0. 453		0.391
Male	174	11.67±5.02		7.25±4.68	
Female	116	11.18±6.01		6.76±4.98	
Age (yr)			0.809		0.449
≥60	141	11.39±5.71		7.27±5.12	
< 60	149	11.55±5.71		6.84±4.48	
Smoking status			0.819		0.466
No	110	11.42±5.33		6.88±4.459	
Yes	180	11.57±5.62		7.33±5.32	
ECOG PS			0.663		0.276
0-1	270	11.51±5.39		6.97±4.71	
2-3	20	10.96±6.02		8.78±5.16	
Response			0.409		0.287
CR+PR+SD	248	11.38±5.56		7.20±4.78	
PD	42	12.14±5.10		6.50±1.11	
Pathological stage			0.267		0.552
Limited stage	176	11.18±5.09		7.19±4.939	
Extensive stage	114	11.91±5.10		6.85±4.62	
Metastases			0.882		0.942
Yes	111	11.53±5.81		7.03±4.70	
No	179	11.44±5.20		7.07±4.87	
SCLC: small cell lung cancer; ECOG: Eastern Cooperative Oncology Group; PS: performance status; CR: complete response; PR: partial response; SD: stable disease; PD: progressive disease.					

**2:** 化疗前后CD4^+^CD25^+^CTLA4及CD8^+^PD-1的表达水平 The level of CD4^+^CD25^+^CTLA4 and CD8^+^PD-1 prior to chemotherapy (baseline) and after the second cycle of chemotherapy (2^nd^ cycle)

	Baseline	2^nd^ cycle	*P*
PD-1/CD8	11.48±5.91	8.74±3.39	< 0.000, 1
CTLA-4	6.94±4.91	5.11±2.60	< 0.000, 1

### 2.4 PD-L1在SCLC外周血及细胞中的分布情况

本研究在SCLC外周血单个核细胞中（图 3A）及T淋巴细胞中（图 3B）均检测到PD-L1极低水平的表达（0.01±0.001）%，但在SCLC细胞系H446细胞中PD-L1高表达（图 3C），并定位在细胞浆及细胞膜中（图 3D）。

**3:**
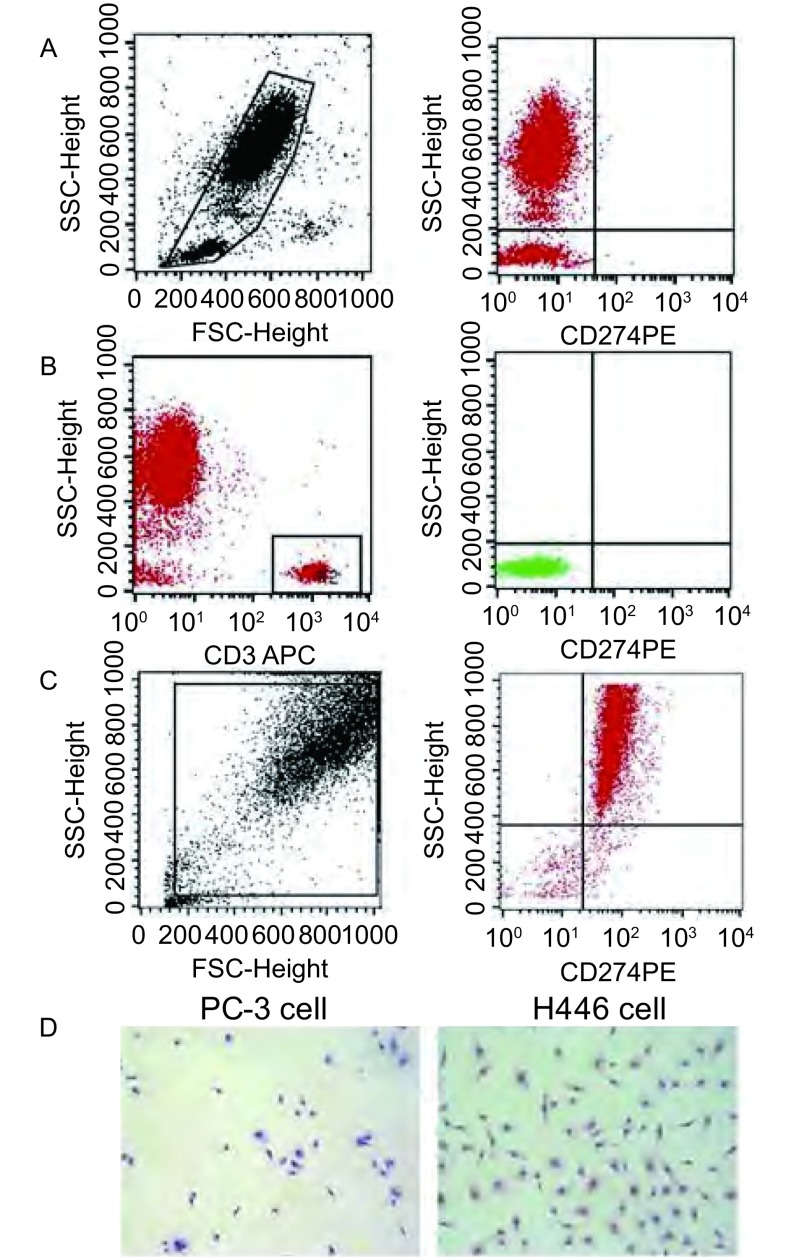
PD-L1在SCLC外周血和H446细胞中的表达及分布。A：左图，外周血单个核细胞群（R1门）；右图，R1门中PD-L1水平；B：左图，CD3^+^ T细胞群（R2门）；右图，R2门中PD-L1水平；C：PD-L1在H446表达（FACS法）；D：PD-L1在H446细胞中表达（ICC法）。PC-3细胞为阴性对照。FACS：流式细胞术；ICC：免疫细胞化学法。 Distribution and expression of PD-L1 in peripheral blood of SCLC patients and H446 cells. A: Left, mononuclear cells in peripheral blood (Gate R1); Right, Level of PD-L1 in Gate R1; B: Left, CD3^+^ T cells (Gate R2); Right, PD-L1 in Gate R2; C: The expression of PD-L1 in H446 cells (FACS); D: PD-L1 expression in H446 cells (ICC) with PC-3 cell as negative control. FACS: flow cytometry; ICC: immunocytochemical method.

## 3 讨论

免疫靶向药物单药或联合用药已经在美国和欧盟批准用于治疗多种晚期实体肿瘤，包括晚期非小细胞肺鳞癌和广泛期小细胞肺癌^[12-14]^，但缺少公认的疗效预测生物标志物，因此难以在治疗前对患者进行筛选和分层进行更精准的治疗^[15]^。

肿瘤组织是进行生物标志物研究和检测的可靠标本来源，但组织活检由于取材创伤性和对活检技术及操作人员要求高，组织存在异质性等，在实际临床诊疗中具有局限性。以血液为代表的液态标本，具有取材便捷无创、不存在异质性、可多次取样以实现动态监测的优点，满足了临床实践需求，因此是临床转化性研究，尤其是肺癌研究领域的热点。在非小细胞肺癌的治疗中使用液态标本进行分子病理诊断得到广泛认可，2016年《中国临床肿瘤学会原发性肺癌诊疗指南》首次将液态标本（循环游离DNA）和液体活检纳入指南中^[16]^。可手术的SCLC患者仅占发病人数的5%左右，肿瘤标本获取更加困难，因此采用血液标本发现新的生物标志物指导SCLC的诊疗更为迫切。

Erfani等^[17]^发现喉鳞状细胞癌CTLA4既高表达在CD8^+^淋巴细胞中，也高表达在CD4^+^和CD19^+^淋巴细胞中，我们的结果显示SCLC患者CD4^+^细胞表达CTLA-4，但CD4、CTLA-4双阳性细胞在SCLC和健康人中表达水平无差异。我们对于CD4细胞进行了进一步的分类，发现SCLC CD4^+^CD25^+^阳性细胞与健康人无差别，但CD4^+^CD25^+^CTLA-4^+^三阳性细胞水平明显高于健康人，平均水平约为3倍，因此我们认为SCLC中CTLA-4可能通过调节性T细胞发挥免疫抑制功能，这与庞春等^[18]^在肝癌中的研究结论一致，该研究认为CD4^+^CD25^+^CTLA-4^+^细胞是一群重要细胞，这类细胞数量的增多可以作为判断侵袭性、进展的一个潜在重要指标。

有关外周血中PD-1在淋巴细胞中的分布及临床意义尚无定论。Waki等^[19]^认为晚期或复发的NSCLC接种合成肽疫苗前后患者外周血中CD4^+^PD-1^+^细胞的含量均与总生存相关，而CD8^+^PD-1^+^细胞在接种疫苗后含量下降与预后总生存延长有关。Kamphorst等^[20]^发现经抗PD-1治疗后70%的患者Ki-67^+^PD-1^+^CD8 T数量升高，该应答是肿瘤特异性的，增殖的CD8^+^T细胞共表达高水平的PD-1和CTLA-4。在本研究中，我们发现SCLC患者CD4和CD8细胞均表达PD-1，但只有CD8细胞中的PD-1水平比健康人低，而CD4细胞中的PD-1水平在两组之间无显著差异，我们认为SCLC外周血中CD8^+^PD-1^+^细胞水平对于预测PD-1免疫靶向治疗可能具有临床价值，该研究与Malaspina等^[21]^在口腔鳞状细胞癌外周血中的发现不一致，她们认为PD-1^+^CD4^+^表达无差异，而CD8^+^PD-1^+^细胞比健康对照组高表达。

本项目还对比了SCLC患者化疗前后CD4^+^CD25^+^CTLA-4^+^和CD8^+^PD-1^+^水平的变化，用以观察治疗疗效与免疫关卡点蛋白表达水平的相关性。结果显示化疗后SCLC CD4^+^CD25^+^CTLA-4^+^细胞和CD8^+^PD-1^+^细胞水平相比治疗前下降，说明该水平可能与肿瘤负荷相关，该结论与Wang等^[22]^的研究结果一致。Wang等^[22]^发现放疗和免疫调节治疗后癌症患者外周血中PD-1的mRNA水平明显升高，联合分析外周血中PD-1、CTLA-4和其他免疫分子可以为抗PD-1或抗PD-L1治疗提供线索。

文献报道PD-L1高表达于SCLC肿瘤组织，我们在SCLC肿瘤组织（免疫组化法）和SCLC细胞系H446（细胞免疫组化和流式细胞术）中均检测到高水平的PD-L1表达。我们采用Cellsearch法在循环肿瘤细胞中检测到PD-L1表达（数据未列），但采用流式细胞术未发现外周血中有PD-L1的表达。这可能是由于外周血中循环肿瘤细胞数目稀少，需要采用比流式细胞术更敏感的方法进行检测，或先需要富集阳性细胞再进行检测。有关PD-L1在外周血中的表达研究报道较少，但Kronig等^[23]^发现Ⅲ期和Ⅳ期黑色素瘤患者外周血Melan-A^+^CD8^+^PD1^+^ T细胞与预后总生存无关，但Melan-A^+^CD8^+^PD-L1^+^的表达与预后有关。

本研究具有一定的局限性，我们仅检测了外周血标本中免疫关卡点蛋白的表达，未检测相应的配对组织标本；患者主要采用化疗，治疗方法单一；本研究为非干预性，临床观察研究，影响因素较多，后续还需要更多随机对照研究进一步证实和验证本研究的结论和发现。

## References

[1] Jahchan NS, Lim JS, Bola B (2016). Identification and targeting of long-term tumor-propagating cells in small cell lung cancer. Cell Rep.

[2] Varghese AM, Zakowski MF, Yu HA (2014). Small-cell lung cancers in patients who never smoked cigarettes. J Thorac Oncol.

[3] Flemming A (2012). Cancer: PD-1 makes waves in anticancer immunotherapy. Nat Rev Drug Discov.

[4] Homicsko K, Duraiswamy J, Doucey MA (2016). Combine and conquer: double CTLA-4 and PD-1 blockade combined with whole tumor antigen vaccine cooperate to eradicate tumors. Cancer Res.

[5] Ribeiro GJ, Schmerling RA, Haddad CK (2016). Analysis of the abscopal effect with anti-PD-1 therapy in patients with metastatic solid tumors. J Immunother.

[6] Tanhapour M, Vaisi-Raygani A, Bahrehmand F (2016). Association between the cytotoxic T-lymphocyte antigen-4 mutations and the susceptibility to systemic lupus erythematosus; Contribution markers of inflammation and oxidative stress. Cell Mol Biol.

[7] Baroudjiana B, Lourencob N, Pagèsa A (2016). Anti-PD-1-induced collagenous colitis in a melanoma patient. Melanoma Res.

[8] Mai TJ, Ma R, Li Z (2016). Construction of a fusion plasmid containing the PSCA gene and cytotoxic T-lymphocyte associated antigen-4 (CTLA-4) and its anti-tumor effect in an animal model of prostate cancer. Braz J Med Biol Res.

[9] Brahmer JR, Tykodi SS, Chow LQ (2012). Safety and activity of anti-PD-L1 antibody in patients with advanced cancer. N Engl J Med.

[10] Topalian SL, Hodi FS, Brahmer JR (2012). Safety, activity, and immune correlates of anti-PD-1 antibody in cancer. N Engl J Med.

[11] Powles T, Eder JP, Fine GD (2014). MPDL3280A (anti-PD-L1) treatment leads to clinical activity in metastatic bladder cancer. Nature.

[12] Reck M, Heigener D, Reinmuth N (2016). Immunotherapy for small-cell lung cancer: emerging evidence. Future Oncol.

[13] Horn L, Reck M, Spigel DR (2016). The future of immunotherapy in the treatment of small cell lung cancer. Oncologist.

[14] Antonia SJ, López-Martin JA, Bendell J (2016). Nivolumab alone and nivolumab plus ipilimumab in recurrent small-cell lung cancer (CheckMate 032): a multicentre, open-label, phase 1/2 trial. Lancet Oncol.

[15] Wolchok JD, Hoos A, O'Day S (2009). Guidelines for the evaluation of immune therapy activity in solid tumors: immune related response criteria. Clin Cancer Res.

[b16] 16Wu YL ed. CSCO guidelines in the diagnosis and treatment of primary lung cancer. Beijing: People Health Publishing House, 2016. V1. 2016: 1-74.吴一龙主编. 中国临床肿瘤学会(CSCO)原发性肺癌诊疗指南2016. V1. 北京: 人民卫生出版社, 2016: 1-74.

[17] Erfani N, Khademi B, Haghshenas MR (2013). Intracellular CTLA-4 and regulatory T cells in patients with laryngeal squamous cell carcinoma. Immunol Invest.

[18] Pang C, Wang HY, Han F (2009). Expression of CD4^+^CD25^+^ Treg cell in hepatocellular carcinoma and its clinical significance. Zhongguo Shi Yong Yi Kan.

[19] Waki K, Yamada T, Yoshiyama K (2014). PD-1 expression on peripheral blood T-cell subsets correlates with prognosis in non-small cell lung cancer. Cancer Sci.

[20] Kamphorst AO, Pillai RN, Yang S (2017). Proliferation of PD-1^+^ CD8 T cells in peripheral blood after PD-1-targeted therapy in lung cancer patients. Proc Natl Acad Sci U S A.

[21] Malaspina TS, Gasparoto TH, Costa MR (2011). Enhanced programmed death 1 (PD-1) and PD-1 ligand (PD-L1) expression in patients with actinic cheilitis and oral squamous cell carcinoma. Cancer Immunol Immunother.

[22] Wang W, Shen G, Wu S (2017). PD-1 mRNA expression in peripheral blood cells and its modulation characteristics in cancer patients. Oncotarget.

[23] Kronig H, Julia Falchner K, Odendahl M (2012). PD-1 expression on Melan-A-reactive T cells increases during progression to metastatic disease. Int J Cancer.

